# Allergen immunotherapy and allergic rhinitis: false beliefs

**DOI:** 10.1186/1741-7015-11-255

**Published:** 2013-12-05

**Authors:** Moisés A Calderón, A William Frankland, Pascal Demoly

**Affiliations:** 1 Section of Allergy and Clinical Immunology, Imperial College London - National Heart and Lung Institute, Royal Brompton Hospital, Dovehouse Street, London, UK; 2 London Allergy Clinic, New Cavendish Street, London, UK; 3 Allergy Division, Pneumology Department, INSERM U657-University Hospital of Montpellier, Avenue du Doyen Gaston Giraud, Montpellier, France

**Keywords:** Allergen, Immunotherapy, Specific, Sublingual, Subcutaneous, Desensitization, Vaccination

## Abstract

**Background:**

Over the last 100 years, several persistent misconceptions or ‘false beliefs’ have built up around allergen immunotherapy and its use in allergic rhinitis. This is perhaps because enthusiastic physicians administered complex allergen extracts to a diverse population of patients suffering from heterogeneous atopic conditions. Here, we review evidence that counters seven of these ‘false beliefs.’

**Discussion:**

1. The symptoms of allergic rhinitis can be more heterogeneous, more severe and more troublesome in everyday life than many physicians believe. Large-scale epidemiological surveys show that the majority of allergic rhinitis patients have at least one symptom severe enough to interfere with sleep quality, productivity and/or well-being. 2. Allergen immunotherapy is not necessarily suitable for all allergic rhinitis patients (notably those with mild symptoms). Recent evidence from double-blind, placebo-controlled, randomized clinical trials suggests that the more severe the disease, the greater the treatment effect. 3. Allergen immunotherapy is often accused of lack of efficacy (relative to pharmacotherapy, for example). However, there are now many meta-analyses, systematic reviews and high-quality clinical trials that find overwhelmingly in favor of the efficacy of allergen immunotherapy (including sublingual formulations) in allergic rhinitis induced by pollen and, increasingly, other allergens. 4. Natural-exposure and challenge-chamber trials have shown that symptom relief may become apparent within months or even weeks of the initiation of allergen immunotherapy. 5. In pollen-induced allergic rhinitis, several years of subcutaneous or sublingual allergen immunotherapy are associated with sustained clinical efficacy after subsequent treatment cessation – confirming the disease-modifying nature of this therapy. 6. Most patients seeking treatment for allergic rhinitis are polysensitized, and allergen immunotherapy has proven efficacy in large, robust clinical trials in these groups. Polysensitization is not a contraindication to allergen immunotherapy. 7. Sublingual allergen immunotherapy is safe for home administration. A recent review calculated that 1 billion doses were administered worldwide between 2000 and 2010 and found that the 11 case reports of anaphylaxis (all non-fatal) corresponded to non-standard practice.

**Summary:**

Modern, evidence-based medicine has generated more than enough robust evidence to remove misconceptions about allergen immunotherapy and allergic rhinitis.

## Background

In 2011, a flurry of events and publications marked the centenary of Leonard Noon and John Freeman's ground-breaking papers in *The Lancet* - the first scientific descriptions of clinically effective allergen immunotherapy (AIT) for grass-pollen-induced allergic rhinoconjunctivitis [1,2]. However, several persistent misconceptions or ‘false beliefs’ have built up around AIT and its use in allergic rhinitis (AR) over the last 100 years. These misconceptions largely arose because of the empirical, poorly standardized clinical research methods that were widely used in this field until the 1950s. In a sense, Noon and Freeman were ahead of their time in describing their research so precisely and elegantly; their reports triggered attempts by enterprising, enthusiastic physicians to administer complex therapeutics to a diverse population of patients suffering from heterogeneous medical conditions [3]. Unsurprisingly, the clinical results were just as heterogeneous. Allergen immunotherapy involves the regular administration of specific, semipurified allergen extracts, with a view to desensitizing the patient's allergic reaction when the sensitizing allergen is subsequently or concomitantly encountered under ‘natural exposure’ conditions. Although the molecular and cellular details of AIT's mechanism of action are still being worked out, the induction of peripheral T cell tolerance by T regulatory cells is a key step in shifting the immune response to an allergen from a ‘T-helper 2’ profile to a tolerogenic ‘T-helper 1’ profile [4]. Historically, a subcutaneous injection every month or two has been the preferred route for the administration of allergen extracts. However, subcutaneous AIT (SCIT) is associated with a low but non-negligible risk of systemic and potentially anaphylactic reactions and other administration routes have been developed (with a recent emphasis on delivery to the sublingual mucosa) in order to improve the safety profile while maintaining efficacy. However, after a hundred years of clinical experimentation, there is little consensus on the optimal regimen for a given allergen extract and administration route (in terms of the frequency of administration, the dose of allergen administered each time, the duration of treatment and, thus, the cumulative dose). Here, we briefly review a number of persistent misconceptions and cite some of the robust medical and scientific evidence that should lay these false beliefs to rest at last.

## Discussion

### False belief #1: ‘Allergic rhinitis is a trivial, homogenous disease’

In fact, AR is under-diagnosed and undertreated, and its symptoms are more troublesome than many physicians (and indeed some patients) believe. In a Europe-wide survey, two-thirds of AR patients reported at least one symptom that is severe enough to interfere with sleep quality, cognitive function, work productivity, school performance, psychosocial well-being or overall quality of life [5]. Poor sleep is a particular problem. In a study in France, 44% of AR patients reported feeling tired after a night’s sleep and were also more prone to anxiety and depression [6]. In another Europe-wide survey, one-third of patients felt irritable and 12% of both persistent and intermittent sufferers suffered from depression [7]. The study also emphasized the association between disease severity and the presence of comorbidities (including potentially life-threatening asthma); one third of the surveyed AR patients had been diagnosed with asthma and three-quarters of the asthma sufferers had moderate-to-severe AR [7]. We acknowledge that state social security systems may no longer be able to bear the full financial burden of AR, as they face other challenges brought about by the economic global crisis and demands linked to rarer but more severe diseases (such as cancer and neurodegenerative disorders), in comparison with which AR is trivial. However, the immediate consequence of trivializing AR is suboptimal treatment and altered well-being and function for a great number of citizens. In a Danish survey, 83% of patients with moderate-to-severe rhinitis were undertreated (that is, they were receiving antihistamines only or even no treatment at all) [8].

### False belief #2: ‘Allergen immunotherapy is indicated in all allergic rhinitis sufferers’

International guidelines and consensus statements (such as those issued by the Allergic Rhinitis and its Impact on Asthma group) [9] make it very clear that AIT is a second-line treatment for use when AR is severe or poorly controlled by appropriate pharmacotherapy or when pharmacotherapy is refused by the patient or induces undesirable side effects. The guidelines also state that AIT is particularly appropriate in patients suffering from moderate-to-severe AR in whom symptomatic treatments are inefficacious, poorly tolerated or not wanted. There is good evidence that AIT is very suitable for highly symptomatic patients. For example, a *post-hoc* analysis of large subgroups of AR patients receiving grass pollen sublingual allergen immunotherapy (SLIT) [10] found that the relative active versus placebo differences in the symptom score were, respectively, 15%, 26% and 37% in investigating centers likely to have low, moderate and high disease activity (as defined by the symptom scores in the placebo-treated groups). In a similar pediatric trial, centers with low, moderate and severe disease activity had relative active versus placebo differences of 10%, 33% and 34%, respectively. Hence, although a treatment effect was seen in patient groups with low initial symptom scores, the magnitude of the effect was greater in groups with higher initial scores. Using a different approach (based on an analysis of the frequency of ‘days with severe symptoms’ in individual patients), Durham *et al*. [11] came to the same conclusion: the more severe the symptoms, the greater the clinical impact of grass pollen SLIT.

### False belief #3: ‘Sublingual allergen immunotherapy is not efficacious in allergic rhinitis’

With so many meta-analyses ([12], for example), in-depth reviews [13,14] and high-quality, well-powered double-blind, placebo-controlled, randomized clinical trials (DBPC RCTs) ([15,16], for example) finding in favor of SLIT in pollen-induced AR, it is hard to see why this false belief persists. The sometimes conflicting results of studies published before the 1980s (often with small study populations and non-optimal trial designs) may have been responsible for lingering doubt as to the efficacy of SLIT and little was then known about AIT's mechanism of action. The efficacy of SLIT in pollen-induced AR is beyond doubt and our knowledge of how AIT may work is now far more robust [4]. We firmly believe that in time, the remaining question marks over the efficacy of AIT in the treatment of AR induced by other allergens (for example, house dust mite allergens) will be dispelled by methodical clinical investigation.

### False belief #4: ‘Allergen immunotherapy does not have short-term clinical benefits in allergic rhinitis’

The fact that several consecutive seasons or years of AIT are recommended may have inspired the misconception that this therapy only becomes effective after several years. Noon and Freeman noted clinical effects after treating patients ‘from a few weeks to eight months’ [1,2,17]. The vast majority of recent, large-scale trials involved two to four months of pre-seasonal treatment before the pollen season in which significant clinical efficacy was observed [12-16]. In a DBPC RCT of patients taking sublingual grass pollen tablets, controlled exposure to pollen outside the season (in an allergen challenge chamber) at treatment initiation and one week and one, two and four months thereafter showed that the active versus placebo difference in symptom score became statistically significant at one month [18]. Hence, SLIT has clinical benefits after just a few weeks of administration.

### False belief #5: ‘There is no sustained effect after discontinuation of allergen immunotherapy’

This misconception is hard to equate with the previous one, since AIT would then have neither short-term nor long-term efficacy. Allergen immunotherapy differs markedly from symptomatic drugs in that it can produce sustained symptom relief after treatment discontinuation [19]. For example, a study involving 257 subjects with grass pollen rhinoconjunctivitis (who had been randomized to three years of daily treatment with grass pollen SLIT tablets or placebo) found that clinical improvements and accompanying immunological changes were sustained for at least two years [20]. In addition to increasing efficacy from one season to another while on AIT tablets, patients showed a similar, sustained reduction in symptom and medication scores one year after treatment cessation (with mean reductions of 26% and 29%, respectively). However, the recent, large-scale clinical trials demonstrating the post-treatment efficacy of grass pollen SCIT and SLIT involved three previous treatment seasons; it appears that sustained treatment is required for sustained post-treatment efficacy and that (with today's allergen formulations, at least) a single season or year of treatment is not sufficient. As with any chronic regimen, AIT thus requires good levels of patient compliance to be efficacious. Furthermore, allergens other than grass pollen remain to be investigated in detail. Nevertheless, AIT is clearly a disease-modifying treatment – something that antihistamines and corticosteroids will never be.

### False belief #6: ‘Allergen immunotherapy is not appropriate in polysensitized patients’

This misconception is only slightly less sensible than saying ‘antihistamines are not appropriate in polysensitized patients.’ Allergen immunotherapy has proven efficacy in large, robust clinical trials in primarily polysensitized patients [21]; it would be hard to achieve such a significant overall treatment effect through high efficacy in a minority of patients. Indeed, *post-hoc* analyses have confirmed that sensitization status was not a significant covariate in placebo-controlled efficacy in two large trials [22,23].

### False belief #7: ‘Home administration of SLIT with inhalant allergens is not safe’

It is absolutely clear that sublingual formulations of inhalant allergens have an excellent safety profile – perhaps the best of any therapeutic used to treat allergic disease. A recent review of 11 case reports of anaphylaxis (all non-fatal) found that none corresponded to standard practice in SLIT [24]; in fact, the events involved non-standardized extracts, rush protocols, overdoses and patients who had previously discontinued SCIT due to serious adverse reactions. The authors calculated that one billion SLIT doses had been administered worldwide between 2000 and 2010 (that is, one case of anaphylaxis per 100 million SLIT administrations or one per 526,000 treatment years). This excellent safety profile may be due to rapidly occurring antigen capture by local, tolerogenic antigen-presenting cells and the low numbers of mast cells in sublingual tissues [24]. Nevertheless, all physicians prescribing allergen immunotherapy (and, indeed, all patients receiving it) should be aware of the risk of anaphylaxis and know how to recognize and treat the condition (or seek treatment) promptly.

### False belief #8: ‘Allergic disease is constant over a patient's lifetime’

There is sound epidemiological evidence to show that the activity of allergic respiratory diseases changes with age and that children develop and/or display atopy within the first years or even months of life [25]. In general, the ‘allergic march’ means that many AR sufferers will develop allergic asthma (AA) (and vice versa). Conversely, some patients will "outgrow" one or more of their allergies (essentially during adolescence) [26]. The chronology of the natural history of AR and AA raises the question of the age at which a disease-modifying treatment, such as AIT, could be introduced in atopic children [27]. Today's guidelines state that for safety and compliance reasons, AIT is only suitable for children over the age of 5. However, in view of the early onset of atopy, we consider than clinical investigations of AIT in under-fives are ethically justified. There is also a need to develop allergy prevention strategies (the best being breastfeeding, at present) and test novel, minimally invasive formulations that are suitable for use in young children (such as allergen patches) and that may facilitate extension of the robust evidence on pollen AIT to food allergies [27].

## Summary

Allergic rhinitis is a heterogeneous, under-diagnosed, undertreated, chronic, allergic respiratory condition. If physicians disregard the severity of symptoms, AR sufferers will remain exposed to co-morbidities and poor quality of life. Leonard Noon and John Freeman's ground-breaking papers in 1911 prompted enthusiastic but empirical and sometimes unethical clinical practice and research in the field of allergen immunotherapy in the first half of the 20^th^ century. As a result, allergen immunotherapy for AR has long suffered from the persistence of ‘false beliefs’ driven by poor methodology. Despite being a guidelines-recommended treatment, AIT is often not considered by the physician. However, there is now more than enough evidence from recent robust DBPC RCTs, meta-analyses and large-scale epidemiological surveys to allay misconceptions about AIT – the only disease-modifying treatment for respiratory allergies. In appropriately screened patients, modern AIT is undoubtedly safe and effective in the treatment of AR induced by common aeroallergens.

## Abbreviations

AIT: Allergen immunotherapy; AR: Allergic rhinitis; DBPC RCT: Double-blind, placebo-controlled, randomized clinical trial; SCIT: Subcutaneous allergen immunotherapy; SLIT: Sublingual allergen immunotherapy.

## Competing interests

Moisés A. Calderón has received consulting fees, honoraria for lectures and/or research funding from ALK, Stallergenes, Merck, Allergopharma, HAL and Allergy Therapeutics. A. William Frankland has received travel grants from Stallergenes. Pascal Demoly is a consultant for Stallergenes, ALK, Circassia and Chiesi and an investigator for Stallergenes, ALK, Pierre Fabre Médicament, Ménarini and was a speaker for Stallergenes, ALK, Ménarini, Allergopharma, MSD, AstraZeneca and GlaxoSmithKline in 2010–2013.

## Authors' contributions

All authors were involved in drafting the manuscript. The authors accept full responsibility for the work and the decision to publish. All authors read and approved the final manuscript.

## Authors' information

This article is the fruit of recent discussions at Dr. A. William (‘Bill’) Frankland's London home. Bill Frankland has been described as the ‘grandfather of allergy’ in the UK, starting in 1946 in the Allergy Department at Saint Mary's, where he notably pioneered the use of the controlled, randomized clinical trial in this area. Bill recently celebrated his 101^st^ birthday and still practices at the London Allergy Clinic (in collaboration with another physician). His long scientific and medical career puts him in a unique position to evaluate passing fads and hard facts. Dr. Moises A. Calderon and Dr. Pascal Demoly are active younger members of the European Association of Allergy and Clinical Immunology (EAACI) and have a particular focus on assessing the quality of research evidence in the treatment of respiratory allergies.

## Pre-publication history

The pre-publication history for this paper can be accessed here:

http://www.biomedcentral.com/1741-7015/11/255/prepub
